# A computational lens for sexual-stage transmission, reproduction, fitness and kinetics in *Plasmodium falciparum*

**DOI:** 10.1186/s12936-016-1538-5

**Published:** 2016-09-21

**Authors:** Mara K. N. Lawniczak, Philip A. Eckhoff

**Affiliations:** 1 Wellcome Trust Sanger Institute, Hinxton, CB10 1SA UK; 2 Institute for Disease Modeling, 3150 139th Avenue SE, Bellevue, WA 98005 USA

**Keywords:** *Plasmodium falciparum*, Gametocytes, Mathematical model

## Abstract

**Background:**

The burden of falciparum malaria remains unacceptably high in much of sub-Saharan Africa and massive efforts are underway to eliminate the parasite. While symptoms of malaria are caused by asexual reproduction of the parasite, transmission to new human hosts relies entirely on male and female sexual-stage parasites, known as gametocytes. Successful transmission can be observed at very low gametocyte densities, which raises the question of whether transmission-enhancing mechanisms exist in the human host, the mosquito, or both.

**Methods:**

A new computational model was developed to investigate the probability of fertilization over a range of overdispersion parameters and male gamete exploration rates. Simulations were used to fit a likelihood surface for data on rates of mosquito infection across a wide range of host gametocyte densities.

**Results:**

The best fit simultaneously requires very strong overdispersion and faster gamete exploration than is possible with random swimming in order to explain typical prevalence levels in mosquitoes. Gametocyte overdispersion or clustering in the human host and faster gamete exploration of the mosquito blood meal are highly probably given these results.

**Conclusions:**

Density-dependent gametocyte clustering in the human host, and non-random searching (e.g., chemotaxis) in the mosquito are probable. Future work should aim to discover these mechanisms, as disrupting parasite development in the mosquito will play a critical role in eliminating malaria.

**Electronic supplementary material:**

The online version of this article (doi:10.1186/s12936-016-1538-5) contains supplementary material, which is available to authorized users.

## Background

**Fig. 1:**
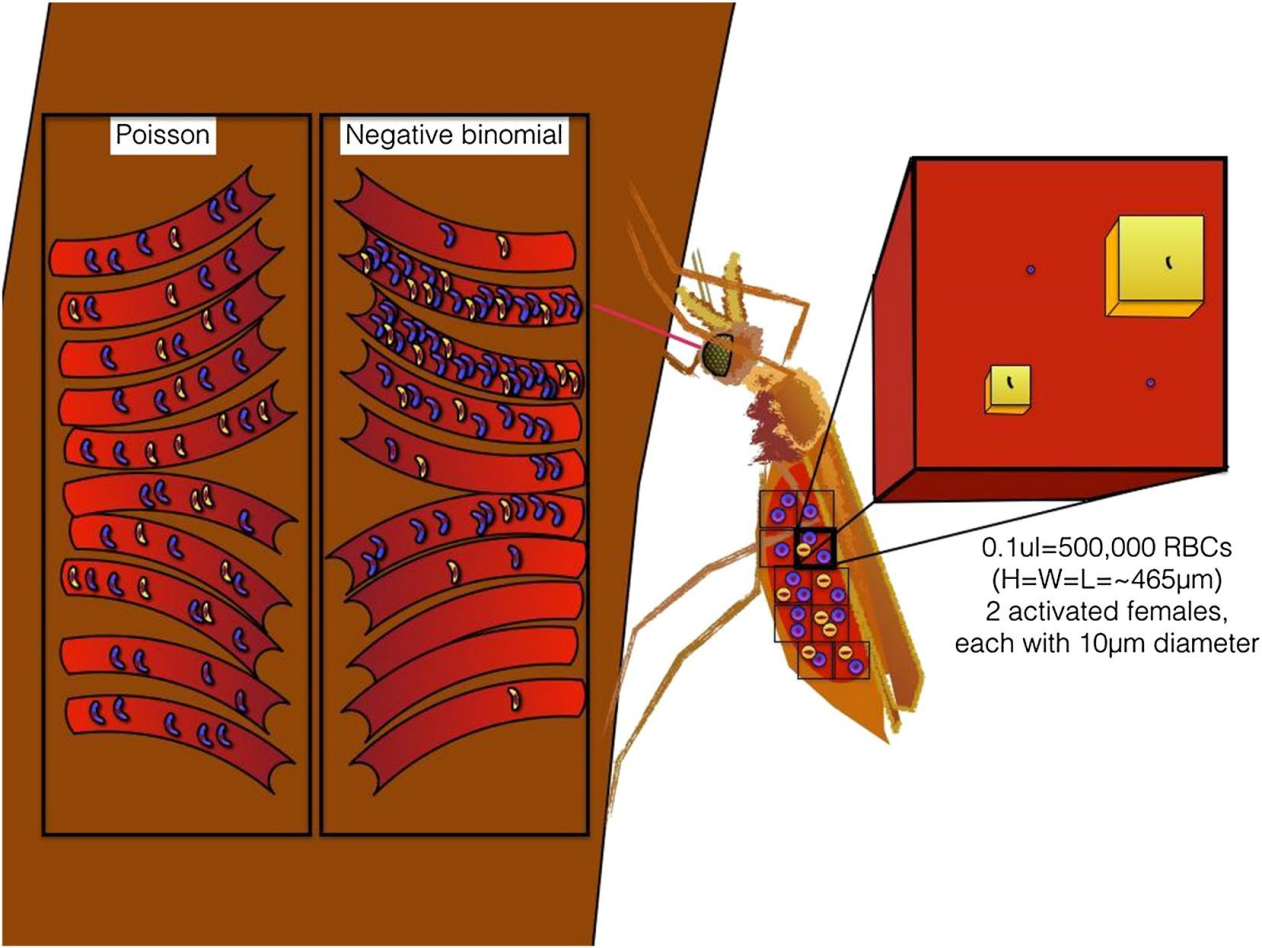
Gametocytes are drawn from Poisson (*left*) or negative binomial (*right*) distributions, with a mean of 5 gametocytes/µl (the threshold of microscopic detection). Each capillary represents exactly 1 µL of blood and in addition to the male (*yellow*) and female (*purple*) gametocytes pictured, also contains 5 million RBCs (represented by the *red* background). Definite failures to infect are in the bottom capillaries, containing no or single sex gametocytes (2 failures for Poisson, 3 for negative binomial). The female mosquito takes her blood meal from the capillary with a relatively high number of males (7) and females (14), represented in her gut. The *red cube* zooms in on 1/10th of this meal (0.1 µl). As noted, this cube contains 500,000 RBCs but only 2 females and 1 male. The *purple dots* in the *red cube* are the approximate relative size of immotile females in 1/10th of a µL, and the two *yellow cubes* represent the volume explored by a single male swimming at 5 µm/s (slow) or 50 µm/s (fast). The male gametocyte present in this cube could produce up to 8 male gametes, but it would take 500 slow males or 50 fast males to fully explore this cube

Aggregation, or clustering, is a common feature of parasites and can clearly influence transmission rates ([1, 2]; Fig. 1). Overdispersion of *Plasmodium falciparum* gametocytes has been observed inside the guts of naturally fed mosquitoes [3, 4] in spite of no physical clustering of gametocytes in circulation ever being reported. Other hints that malaria parasites might employ transmission-enhancing strategies exist. For example, mosquitoes fed directly on skin are more likely to get infected than mosquitoes fed via membrane on blood from the same donor [5]. While this could be due to loss of infectivity during the process of drawing venous blood and setting up membrane-feeding assays [6], it could also be due to different numbers of accessible or mature gametocytes in these different compartments of circulating blood. Some older literature suggests there may be more gametocytes in skin capillaries than in circulating blood, further hinting at gametocyte sequestration in the skin, although these experiments were not done with proper controls [7, 8]. Additional support that *P. falciparum* employs transmission-enhancing strategies comes from membrane feeds using donor blood containing sub-microscopic gametocytaemias (<5/μl); mosquitoes are regularly infected in such experiments [9]. Although sub-microscopic carriers are less infectious than those with higher gametocyte densities, collectively they constitute non-trivial fractions of the infectious reservoir [9]. Once in the midgut, gametes exit the human red blood cell membrane and are faced with the improbable task of finding each other amidst millions of red blood cells (Fig. 1). Altogether, these findings suggest that parasites must be very good at finding each other in both the human and the mosquito.

In order to investigate overdispersion, gamete exploration of the blood meal, and other mechanisms that could influence parasite fertilization rates in the blood meal, a new computational model was developed as described in the “Methods” section. The resulting model generates a distribution for the number of zygotes formed per feed under given conditions and parameters, as well as the fraction of feeds with at least one zygote. Model outputs to experimental data for oocyst counts and prevalence (i.e., fraction of mosquitoes infected) were compared over a wide range of gametocyte densities [10, 11]. It is challenging to fit these data and others [12–14] because models based on random Poisson draws tend to underpredict success at low gametocyte densities and either rise too rapidly towards complete success or plateau artificially at a lower success rate. The new model structure allows for the inclusion and exploration of transmission-enhancing mechanisms such as aggregation and bloodmeal exploration.

## Methods

The key model parameters are gametocyte density *g* in the human host, female sex ratio *f*, viable male gametes per male gametocyte *m*, blood meal size *b*, overdispersion parameter *k*, and blood meal coverage per male gamete *c*.

For Poisson gametocyte draws, a Poisson draw for female gametocytes *X* is made with mean *bfg* and a Poisson draw for male gametocytes *Y* with mean *b(1* − *f)g*. For negative binomial draws, a single draw for number of gametocytes is made with mean *bg* and overdispersion parameter *k*. Each gametocyte then has probability *f* of being female and a series of random draws results in the number of female and male gametocytes in that blood meal, represented by *X* and *Y*, respectively.

Within the blood meal, the number of male gametocytes is multiplied by *m* (range 0–8) to get the number of male gametes in the blood meal. The female gametocytes are assumed to be uniformly distributed throughout the volume, although if female and male gametocytes cluster in the human host and then retain proximity in the blood meal, that would facilitate a higher effective blood meal coverage per male gamete.

The blood meal coverage *c* can be estimated through various means. The swimming speed of exflagellated male gametes in ookinete culture medium has been measured as 5 µm/s, which is only sufficient to map out 0.0001 of a 2 µL blood meal in 30 min [15]. This is due to the motion-orthogonal oscillation exploring a 5-µm diameter, which carves out an explored cylinder at 5 µm/s. Thirty minutes of independent exploration at this rate corresponds to approximately 0.0001 of a 2 µL blood meal. Wilson et al. also observed that the wave speed down the male gamete is a factor of ten faster (50 µm/s) with a wavelength similar in dimension to a red blood cell (RBC), so if the male gamete could move at wave speed in the RBC-dense environment of a blood meal, it could potentially explore up to 0.001 of the blood meal in 30 min. Coverage values above 0.001 would tend to mean that effective coverage rates were occurring faster than wave swimming speed, which would invoke alternative mechanisms such as chemotaxis or conserved spatial proximity.

**Fig. 2:**
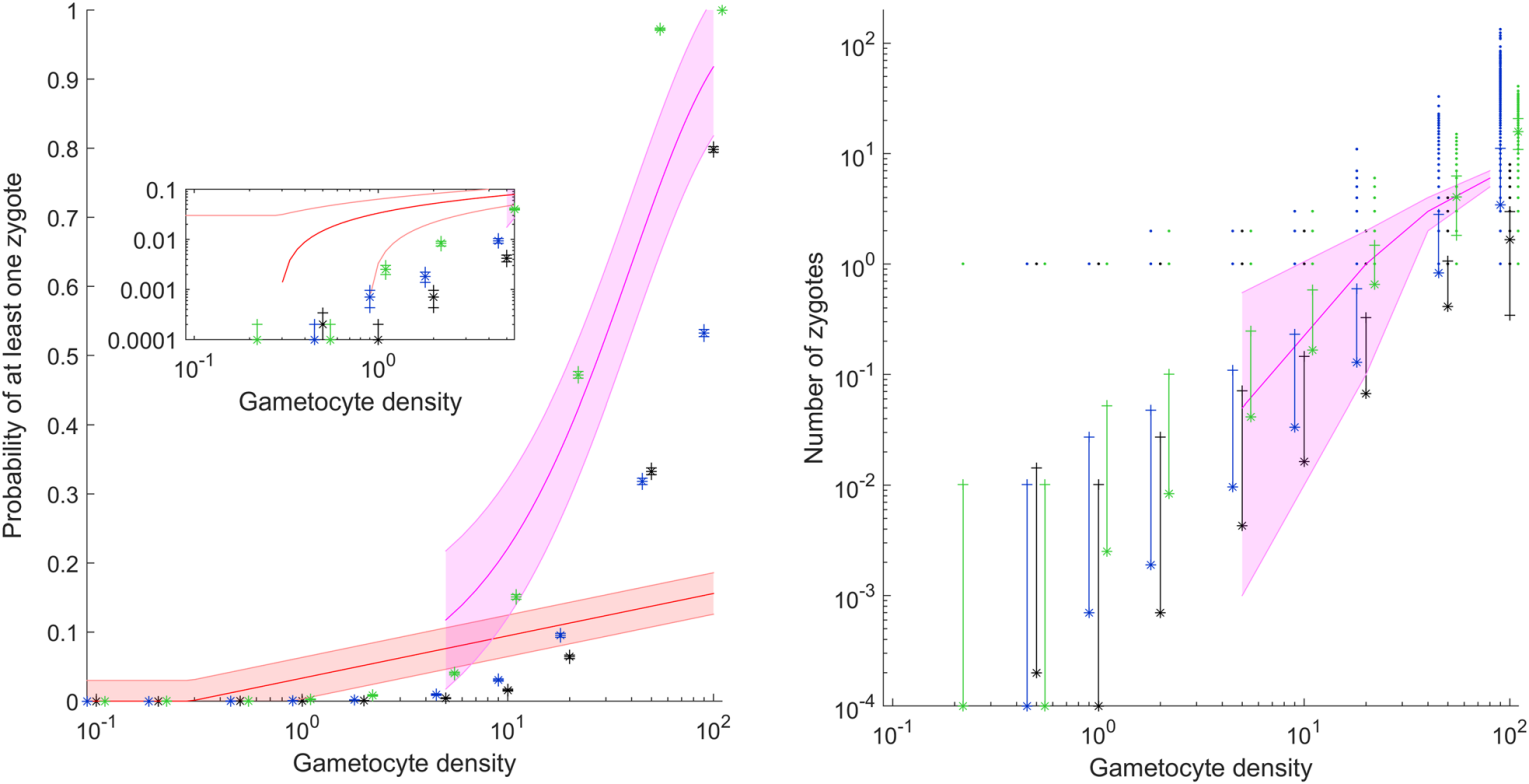
(*Left*) Probability of at least one zygote for varying gametocyte densities, a female sex ratio of 0.7, and 2 male gametes per gametocyte. *Black* is for Poisson gametocyte draws with male gametes exploring 1/10,000 of a blood meal, *green* is for Poisson gametocyte draws with male gametes exploring 1/1000 of a blood meal, and *blue* is for an negative binomial gametocyte draw with overdispersion k = 1 and male gametes exploring 1/10,000 of a blood meal. The *red shaded bar* is the Schneider et al. [10]. fit to data, and the pink shaded bar is an approximation to the Da et al. data. The *inset* shows the probability of at least one zygote zoomed in at low gametocyte densities. (*Right*) The distribution, mean and one standard deviation for the number of zygotes for each of the three scenarios simulated on the left. The *pink shaded bar* is for the Da et al. [11] data on oocysts. The histograms for female gametocytes, male gametocytes, and zygotes in a blood meal for each condition in this Figure can be found in Additional file 1: Figures S1–S3

Given a value of *c*, each successfully exflagellated male gamete independently explores the fraction *c* of the blood meal, so the total fraction of volume explored becomes *P* = *1* − *(1* − *c)*^*mY*^. Each female gamete then has probability *P* of being fertilized, and a final set of random draws determines the number of zygotes. Additional file 1: Figures S1–S3 show histograms for female gametocytes in a blood meal, male gametocytes in a blood meal, and successful zygotes for each of the three conditions in Fig. 2 for various gametocyte densities.

For the varying-*k* model, *k* = *0.5* for gametocyte densities below 1/µL, and then *k* = *0.5* + *0.5* *log*_*10*_*(g)* above 1/µL.

The probability of mosquito infection at low gametocytaemias has been fit previously using experimental infections [10], and the equations describing the fits were used here, with a standard deviation of 0.01. The probability of successful infection and number of oocysts at higher gametocytaemias using experimental infections [11] were manually digitized here from Fig. 1 in the manuscript, with standard deviations taken from the error bars for probability of infection. These previously published data show a steady increase in infection success with gametocyte density, rising above 80 % by 80 gametocytes/µL, with an infection rate of almost 8 % down at the microscopic threshold of 5 gametocytes/µL.

For each set of parameters, 10,000 mosquito feeds were simulated for each of various values of gametocyte densities—0.1, 0.2, 0.5, 1, 2, 5, 10, 20, 50, and 100 gametocytes/µL. The fit quality was Σ −*(simulation mean*-*data mean)*^*2*^*/(data std dev)*^*2*^ for each density, with Schneider [10] data used for gametocyte densities of 4 or less and Da [11] data used for gametocyte densities of 4 or more.

## Results

### Likelihood of zygote formation under different overdispersion scenarios and male gamete swimming speeds

Figure 2 (black) shows the results for a Poisson draw of male and female gametocytes, with male gametes exploring the blood meal at measured free solution swimming speeds, as estimated in [15]. Infection success is negligible below 10 gametocytes/µL, after which fertilization increases rapidly to over 80 % success by 100 gametocytes/µL. This baseline model fails to capture the non-trivial infection rates achieved by sub-microscopic gametocyte densities [10]. Note that since the model outputs zygote counts, these should necessarily be higher than experimental oocyst counts. Previous work counting gametocytes inside mosquito guts estimated overdispersion in blood meals ranging from k < 1 to k = 3, with k = 3 the best overall value [3] and from k = 0.5 at low gametocyte densities to approximately k = 3 at high gametocyte densities [4]. Such overdispersion values are far from Poisson-distributed. When overdispersion k = 1 is used in the model (Fig. 2, blue), success relative to baseline increases at low gametocyte densities and decreases above 50 gametocytes/µL, but the increase at low densities remains below observations [10]. This strong overdispersion of gametocyte draws, such as would come from within-host clustering of gametocytes, reshapes the infection success curve to be much more like experimental data, but computed zygote counts remain well below experimental oocyst counts, instead of being substantially higher. This is problematic given that 100+ fold [16] to 1000+ fold [17] reduction in parasite numbers are estimated in the transition from gametocyte to oocyst.

If male gametes travel at their oscillatory wave speed as described in “Methods” section, blood meal coverage per gamete is multiplied by ten. For a Poisson draw of male and female gametocytes and this faster exploration, fertilization rates rise uniformly over baseline (Fig. 2, green). However, infection success remains far too low at sub-microscopic gametocyte densities, infection rates rise too rapidly above 50 gametocytes/µL, and below 50 gametocytes/µL zygote densities are below oocyst data.

### The formation of zygotes requires more than one explanatory factor

**Fig. 3:**
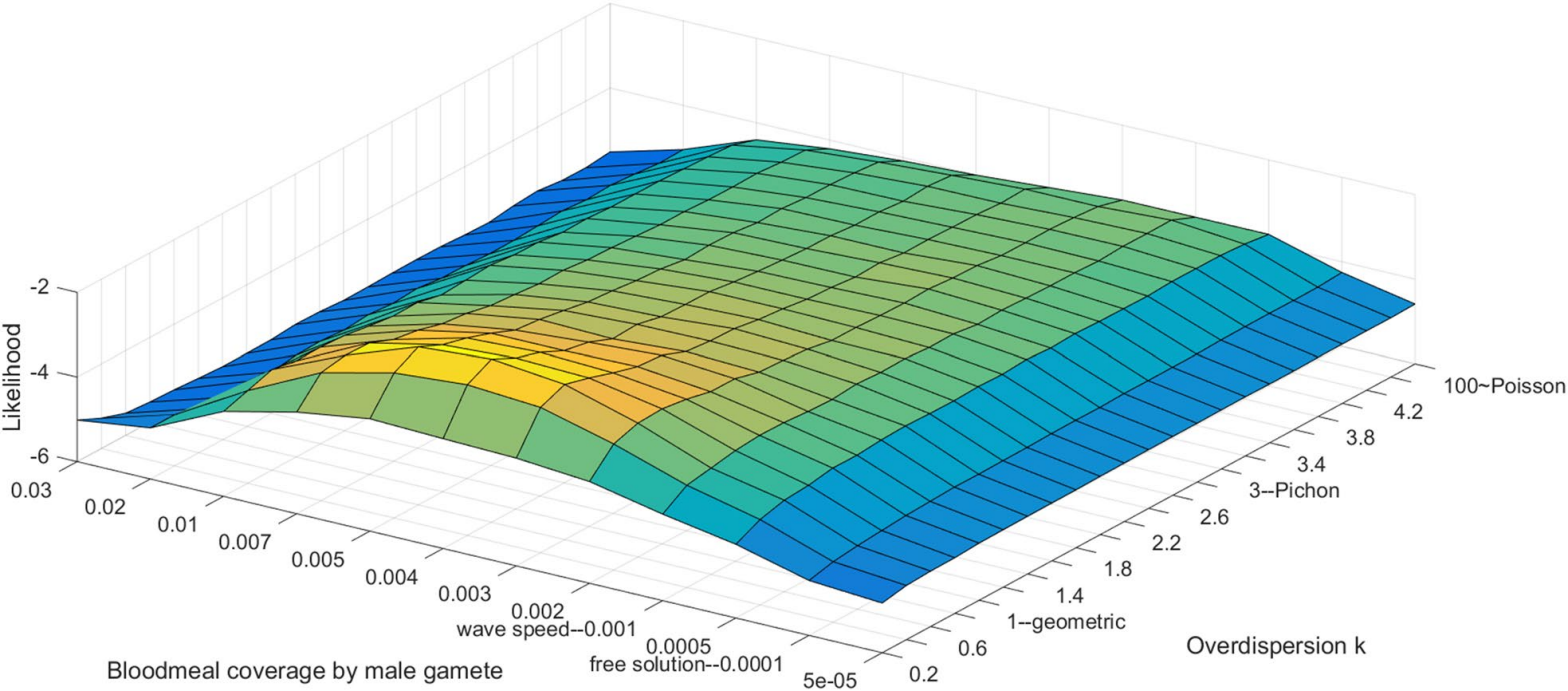
Mapping the likelihood of model fitting to Schneider et al. [10] and Da et al. [11] data for varying overdispersion parameter k and blood meal coverage per male gamete. A clear likelihood peak is seen in both dimensions showing that both more efficient exploration of the blood meal and strongly overdispersed clustering are both needed to get the best fit

A previous model identified density dependence of the clustering parameter, k, such that extreme overdispersion was required at the lowest gametocyte densities [4]. In the model presented here, high overdispersion (k = 1) alone is unable to explain the number of zygotes that must form. Similarly, increasing male gamete speed from 5 to 50 µm/s is insufficient to explain infection success at low densities. One way to explore more area than possible via the fastest random exploration is to explore non-randomly, and within-blood meal chemotaxis or preserved spatial proximity of clustered gametocytes once they have entered the gut could achieve effective blood meal coverages over 0.001 per gamete. Likelihoods of recreating experimental data were calculated as described in “Methods” section, varying overdispersion from k = 0.2 to Poisson-like values and blood meal exploration from 0.00005 to 0.03, with Fig. 3 plotting the resulting likelihood surface.

The likelihood surface in Fig. 3 shows that both fast exploration (coverage > 0.001) and strong overdispersion (k < 1) are jointly favoured, with best values approximately k = 0.6 and coverage = 0.004. Reduced overdispersion, regardless of blood meal coverage, results in lower likelihoods, as do all physiologically possible random swimming speeds independent of clustering. This strongly suggests that at least two separate mechanisms are responsible for observed transmission success at low gametocyte densities.

**Fig. 4:**
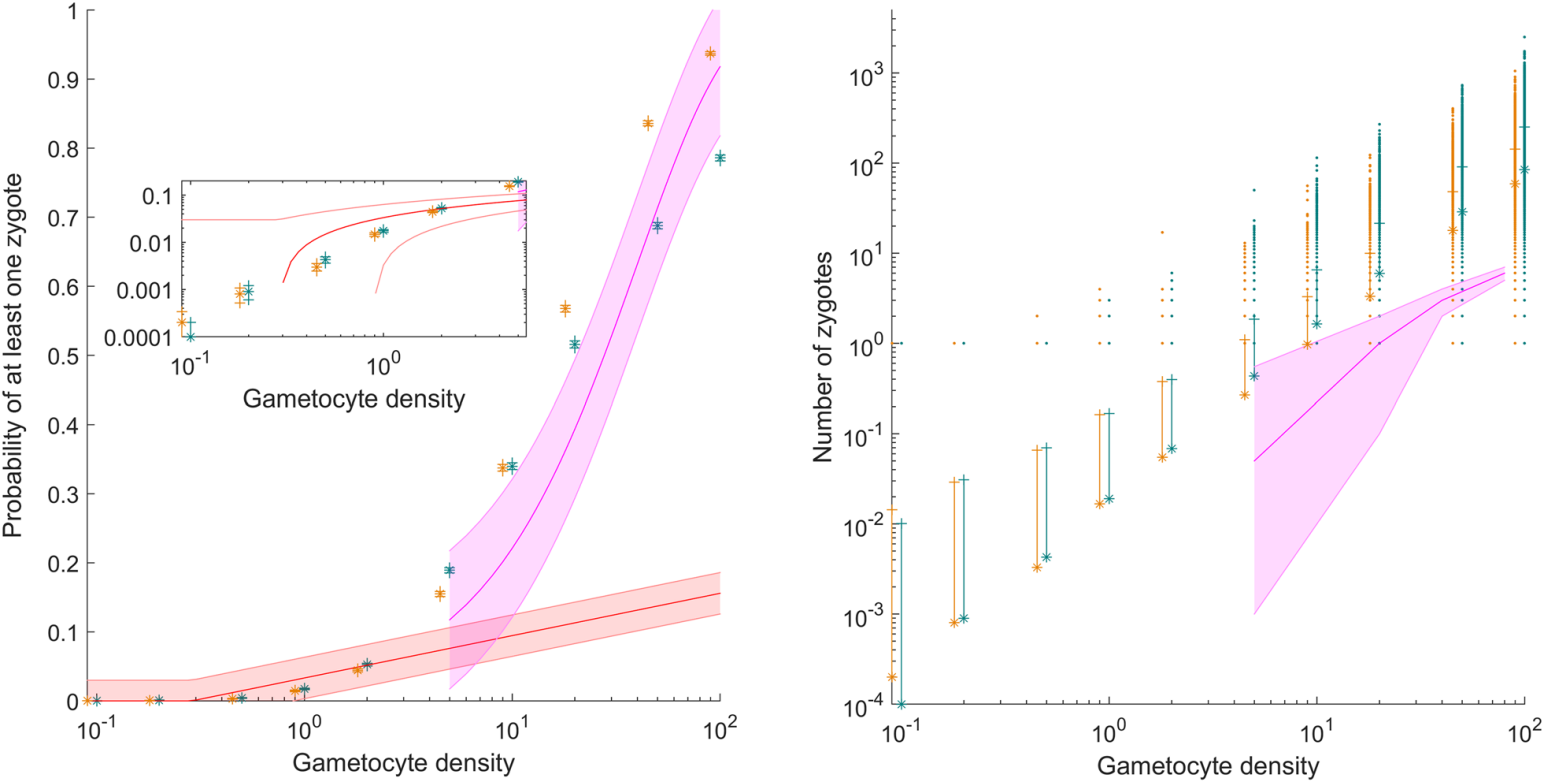
(*Left*) The probability of at least one zygote for the best fitting model parameters (k = 0.6 and blood meal coverage by male gamete of 0.004) is shown in *blue*–*green*. Combining the clustering and rapid blood meal search mechanisms creates a close approximation to the Schneider et al. [10] and Da et al. [11] data across all density regimes studied. If the overdispersion parameter k is allowed to vary from 0.5 at low densities, increasing above a gametocyte density of 1/µL at 0.5 per log10 of density, then an even better fit is achieved, as seen in orange for male gamete blood meal coverage of 0.003. (*Right*) The zygote distributions follow a similar line as the Da et al. [11] data, although the fitting process did not fit to oocyst number. As desired, the mean number of zygotes is almost a full order of magnitude higher than the oocyst counts, with the divergence increasing at higher densities. Note that the varying k plot allows a closer fit to the probability of successful infection while reducing the excessively high zygote counts at the top of the distribution for 100 gc/μl

The best-fitting parameters from the likelihood plot (k = 0.6, coverage = 0.004) are simulated in the model, with the resulting infection success rates and zygote counts plotted in Fig. 4 (blue–green). At low densities, infection success follows the sub-microscopic density data [10] closely, then gradually rises towards higher success rates with a slope similar to success rates observed from higher gametocytaemias [11]. Notably, oocyst counts were not used in fitting, only infection success. The resulting zygote distributions means follow a similar trajectory to the oocyst counts, but higher by almost an order of magnitude.

Finally, letting *k* vary as described in “Methods” section and previously observed [4] results in the best likelihood value observed. The maximum likelihood for this function of *k* corresponds to a gamete coverage of 0.003, and the resulting zygote distributions and success rates are seen in Fig. 4 (orange). The fit is improved, and mean zygote counts remain about an order of magnitude above the experimental oocyst data, but the distribution at high gametocyte densities does not rise to such extreme outlier values as the constant-*k* best fit. This makes sense as an optimal strategy for the parasite, as strong overdispersion at low gametocyte densities increases the fraction of mosquitoes infected, while less overdispersion at high densities increases transmission success while reducing the parasite load in the most infected mosquitoes.

Given that gametocyte sex ratio varies and male gametocytes can produce 0-8 viable gametes, sensitivity was explored, while holding other parameters at best-fit values from Fig. 3. The results are seen in Additional file 1: Figure S4: rather than a single likelihood peak, there is a ridge of likely parameter values from moderate female sex ratios of 0.5–0.75 and two male gametes, to higher female sex ratios with higher numbers of male gametes. Given that there is known female bias in gametocyte counts and expected numbers of male gametes tend to be two to three, this likelihood ridge matches observations. Finally, male gametes versus coverage for the varying-*k* optimal fit were varied in Additional file 1: Figure S5. There is a likelihood ridge conserving blood meal exploration: two male gametes with coverage = 0.003 is approximately as likely as six to seven male gametes and coverage = 0.001.

Presence of a likelihood peak in both dimensions of Fig. 3 was robust to sex ratio, male gametes per gametocyte, and the likelihood function, although the optimal values of *k* could vary from 0.6 to 1.2 and the optimal values of *c* could vary from 0.001 to 0.006 depending on the assumptions. In each set of conditions, the conclusion of both mechanisms being jointly favoured is conserved.

## Discussion

Aggregation has often been cited as a likely phenomenon to explain infection rates of mosquitoes [4, 18] yet it still has not been adequately explained from a mechanistic standpoint. Beyond the need to discover how gametocytes carry out aggregation, the model presented here shows that aggregation on its own still remains insufficient to explain realistic probabilities of zygote formation. Additionally, the space that a male gamete can explore under the fastest realistic swimming speeds [15] is also shown to be insufficient. Results presented here show that in order to explain natural rates of infection at low gametocytaemia, gametocytes must use both a within-host clustering mechanism such as cytoadherence-style aggregation and at least one mechanism that increases fertilisation rates in the bloodmeal such as chemotaxis. Cytoadherence is common among different stages of malaria parasites: RBCs infected with asexual stage parasites can adhere to uninfected RBCs (rosetting) and to endothelial cells (sequestration) [19], egressed male gametes stick to RBCs in species-specific manners [20], and immature gametocytes also sequester through unknown mechanisms in the bone marrow [21]. Although circulating mature gametocytes are more deformable than sequestered immature gametocytes [22], it is possible that mature gametocytes also have the capacity to adhere, either to capillary surfaces or to each other as they pass through the capillaries. The shape and/or buoyancy of gametocytes might also influence their movement through capillaries and thus their uptake by mosquitoes. Once gametes egress from the RBC, the results presented here suggest that additional mechanisms are required to reach infection levels observed in nature. *Plasmodium berghei* male gametes have recently been shown to have some chemotaxis towards female gametes [23]. In *P. falciparum,* chemotaxis has not yet been observed but gametes in the mosquito midgut form sticky nanotubes that could facilitate sex either through cytoadherence or chemical gradients [24]. It is also possible that spatial proximity of gametocytes in the host circulation could be maintained after entering the mosquito, or that the process of feeding and diuresis creates dynamics in blood movement inside the mosquito’s gut that brings gametocytes closer to each other inside the mosquito’s gut. Flow dynamics have been observed for broadcast spawners and can greatly enhance fertilization rates in seemingly improbable situations [25].

## Conclusions

Gametocytes are responsible for transmission of *P. falciparum* from humans to mosquitoes, and they can successfully infect a mosquito even at sub-microscopic densities in the human host [10]. Existing models have difficulty capturing the functional form of the rate of mosquito infection *versus* gametocyte density, and mechanistic models with non-clustered draws of gametocytes and random searching of the blood meal cannot reach the infection levels observed in existing data. In order to explore the existence of possible transmission-enhancing mechanisms, a new mechanistic model of sexual-stage transmission was developed and explored here. Both high degrees of clustering in the human host and faster than the fastest possible random exploration in the mosquito blood meal are independently required in order to recreate existing data on mosquito infection. These results strongly indicate that transmission-enhancing mechanisms are likely to exist, while providing estimates of their nature and magnitude.

The present work examines the probability of successful gamete fertilization in the mosquito midgut for varying gametocyte densities at different fixed values of sex ratio, male gametes per gametocyte, and bloodmeal volume. The average infectiousness over the course of a single infection would be influenced by time-varying parasite densities [12] as well as possible variations in parasite sex ratio [26, 27]. The infectiousness over a local human population would depend on local transmission intensity and seasonality [9], with individual infectiousness influenced by age and prior exposure effects on immunity [28]. Future work could investigate the net effect of these variations over the course of an infection in different transmission settings, as well as incorporating the effect of transmission-blocking immunity [9] to expand the modelled range of gametocyte densities.

Exploring the behaviours of these fascinating cells likely has practical relevance. Gametocytes are extremely difficult parasite stages to access due to their low numbers, yet they are absolutely critical for malaria elimination campaigns. Discovering how the sexual stages of these deadly parasites find each other, both in the human host and in the mosquito, will improve human ability to disrupt this process and thus break the cycle of transmission.

## Additional file

10.1186/s12936-016-1538-5 Additional figures.
