# Supplementary material for: A computational lens for sexual-stage transmission, reproduction, fitness and kinetics in Plasmodium falciparum
Source: Malar J. 2016 Sep 21;15:487. doi: 10.1186/s12936-016-1538-5 (PMC5031309; doi:10.1186/s12936-016-1538-5)
Supplement: Supplementary file 1 — 10.1186/s12936-016-1538-5 Additional figures. [file 12936_2016_1538_MOESM1_ESM.pdf]

Supplemental Material for A computational lens for sexual-stage transmission, reproduction, fitness and kinetics in *Plasmodium falciparum*

Mara K. N. Lawniczak and Philip A. Eckhoff

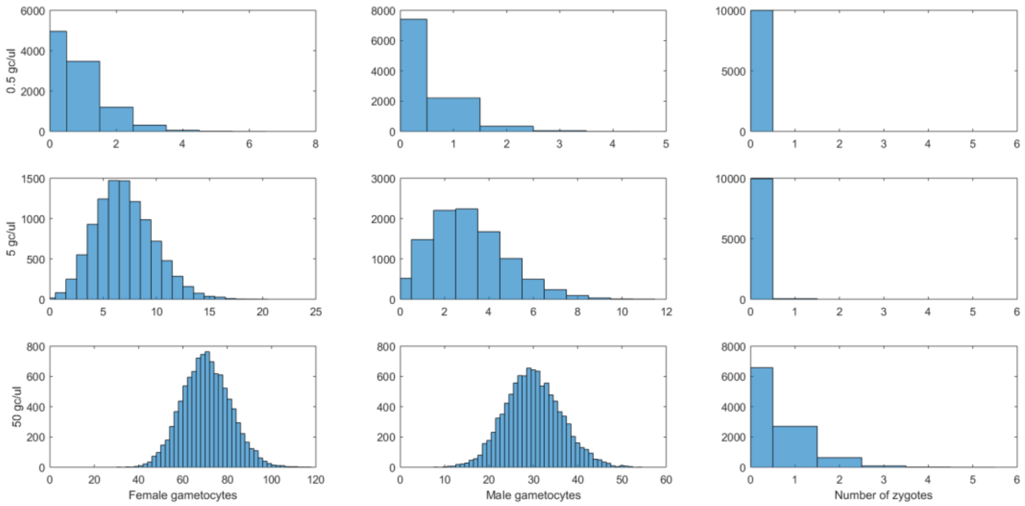

Figure S1: Histograms for female gametocytes, male gametocytes, and zygotes for Poisson draws with male gamete bloodmeal coverage of 0.0001 (black series in Figure 2).

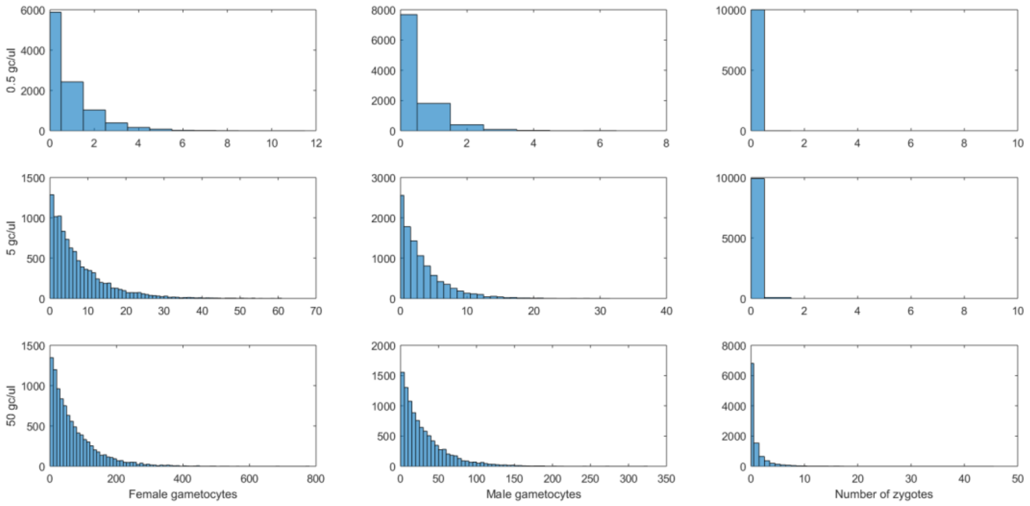

Figure S2: Histograms for female gametocytes, male gametocytes, and zygotes for negative binomial draws with male gamete bloodmeal coverage of 0.0001 (blue series in Figure 2).

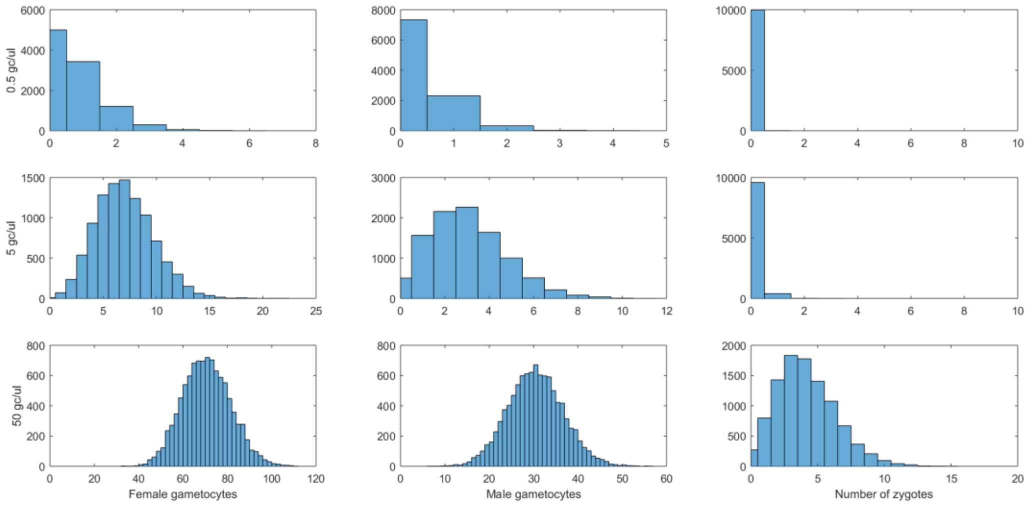

Figure S3: Histograms for female gametocytes, male gametocytes, and zygotes for Poisson draws with male gamete bloodmeal coverage of 0.001 (green series in Figure 2).

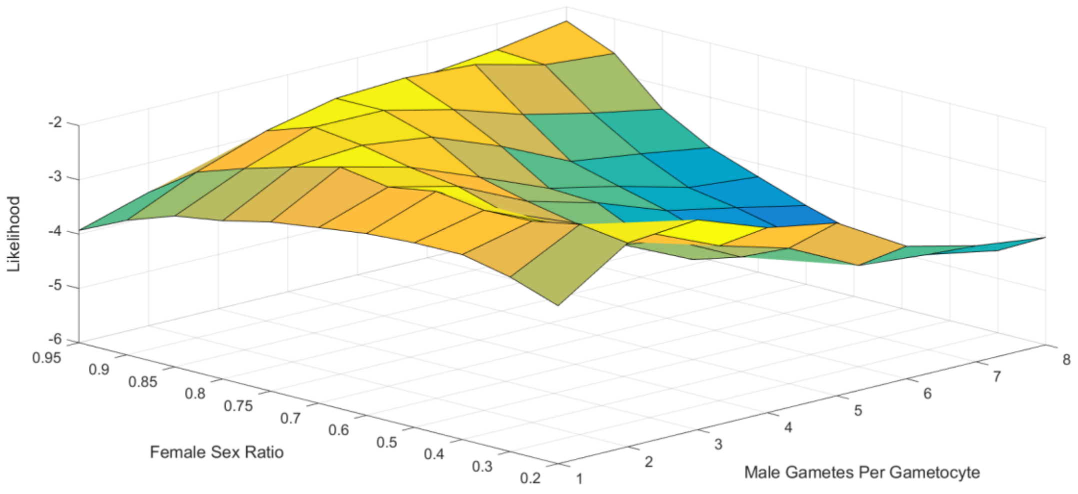

Figure S4: Varying female sex ratio versus male gametes per gametocyte for the best fitting model (k=0.6, coverage=0.004, blue-green in Figure 4) shows a likelihood ridge starting at 2 gametes per gametocyte for moderate sex ratios and increasing as the female sex ratio increases.

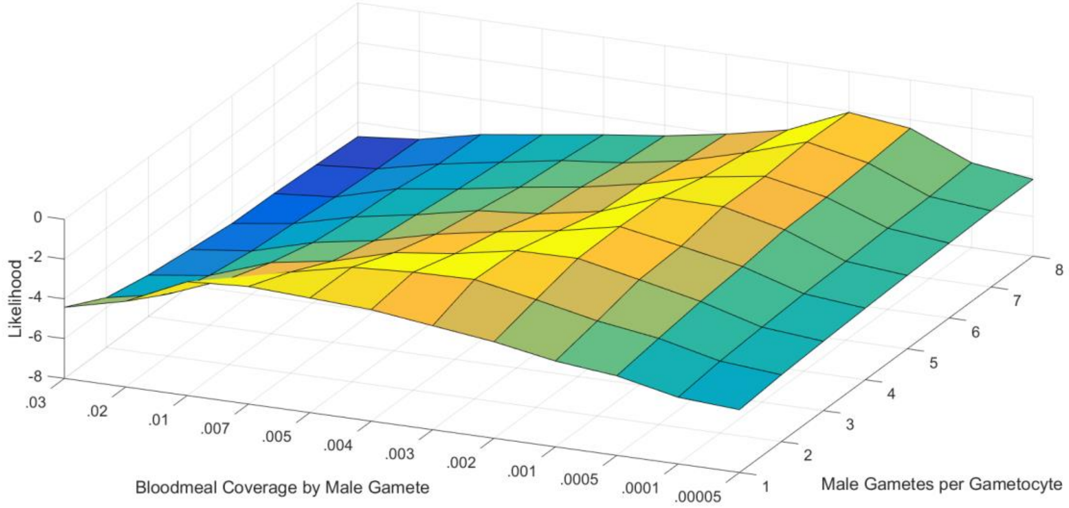

Figure S5: Varying bloodmeal coverage versus male gametes per gametocyte for the varying-k best fit in Figure 4 (orange). As male gametes per gametocyte increases, the required bloodmeal coverage by male gamete to achieve the best fit decreases approximately proportionally.
